# Osteocalcin is associated with triglyceride glucose index rather than HOMA-IR in men with type 2 diabetes

**DOI:** 10.3389/fendo.2022.1067903

**Published:** 2022-12-19

**Authors:** Huijie Huang, Ai Wang, Li Cong, Yingjuan Zeng

**Affiliations:** Department of Endocrinology and Metabolism, The Fifth Affiliated Hospital of Sun Yat-sen University, Zhuhai, China

**Keywords:** osteocalcin, insulin resistance, triglyceride glucose index, HOMA-IR, diabetes

## Abstract

**Introduction:**

The involvement of osteocalcin in the regulation of glucose tolerance in humans is controversial. We utilized a novel and practical insulin resistance surrogate, the triglyceride-glucose (TyG) index, to investigate the association between serum osteocalcin and insulin resistance in men with type 2 diabetes (T2D).

**Methods:**

This was a retrospective cross-sectional study that included 667 male patients suffering from T2D, with measurements of N-terminal mid-fragment of osteocalcin (N-MID), triglycerides (TG), fasting blood glucose (FBG) and C-peptide collected on the same day. We used the TyG index and HOMA-IR as surrogate measures for insulin resistance. Binary logistic regression models that adjust the sociodemographic characteristics and metabolism-related factors were used to assess the associations between osteocalcin and insulin resistance. Restricted cubic spline (RCS) analysis was used to test the potential non-linear relationship between N-MID and the risk of severe insulin resistance. Subgroup analysis evaluated the robustness of the association.

**Results:**

N-MID was correlated with the level of insulin resistance when quantified by the TyG index in unadjusted and adjusted binary logistic regression models (all p < 0.05), but the relationship was not observed when assessed by HOMA-IR (all p > 0.05). RCS model further confirmed that the association between N-MID and the severe insulin resistance measured by the TyG index was non-linear (P = 0.047). Subgroup analysis showed that the association was detected only in younger patients with lower BMI and poorer glycemic control, without hypertension or smoking.

**Conclusions:**

Osteocalcin was inversely associated with the TyG index in men with T2D.

## Introduction

Osteoporosis and fragility fractures, a skeletal complication associated with type 2 diabetes, are of increasing concern recently because of high morbidity, high medical expenses, impairment of quality of life in the elderly, disabling and lethality (1). On the Chinese mainland, one-third of patients with T2D were vulnerable to osteoporosis according to a meta-analysis of fifty-four studies (2). Bone turnover is an indispensable physiological activity for maintaining bone health, which means that worn bone tissue is continuously replaced by newly synthesized calcified bone matrix throughout life (3). Bone turnover markers (BTMs), including osteocalcin, C-terminal telopeptide (CTX), and N-terminal pro-peptide of type-I procollagen (PINP), are used as indicators of bone formation and bone loss, as a means of diagnosing secondary osteoporosis, or assessing the effectiveness of osteoporosis treatment (4). A more noteworthy aspect is that BTMs have been shown to have complex interactions with energy metabolism, which has led to the recognition of bone as an endocrine organ (5). Among these BTMs, osteocalcin is the predominant contributor to influence energy metabolism, mainly through increased secretion of insulin and adiponectin from pancreatic β cells and adipocytes respectively (6), which was demonstrated by a series of animal and cell-based experiments. Lee et al. (7) reported that mice lacking osteocalcin exhibited metabolic abnormalities such as reduced insulin secretion, reduced β cell proliferation and insulin resistance. In addition, the administration of recombinant osteocalcin increased insulin secretion and improved glucose tolerance in wild-type mice, and corrected the manifestation of these metabolic abnormalities in osteocalcin-deficient mice (8). However, studies to analyze whether osteocalcin is relevant to insulin resistance and energy metabolism in humans have yielded inconsistent results (9–15).

Insulin resistance is typically defined as decreased sensitivity or responsiveness to metabolic actions of insulin, such as insulin-mediated glucose disposal and inhibition of hepatic glucose production. Insulin resistance plays a major pathophysiological role in type 2 diabetes and is tightly associated with the globally increased incidence and prevalence of major public health problems, including obesity, hypertension, coronary artery disease, dyslipidemias, and a cluster of metabolic and cardiovascular abnormalities that define the metabolic syndrome (16, 17). Poor glycemic control and an adverse prognosis are often clinical problems for patients with severe insulin resistance. Therefore, it is of great importance to analyze the mechanisms that regulate and ameliorate insulin resistance. Determining the relationship between osteocalcin and insulin resistance in humans may drive the development of osteocalcin as a pharmacological treatment to alleviate the severity of insulin resistance and insulin resistance-related diseases.

Approaches for measuring insulin sensitivity and resistance *in vivo* are various. The triglyceride-glucose (TyG) index, calculated from fasting triglyceride and fasting glucose concentrations, has been considered an indicator that can assess insulin resistance simply and reliably compared with the hyperinsulinemic-euglycemic clamp (HEC) (18). The Homeostasis Model Assessment of Insulin Resistance (HOMA-IR) is currently the most frequently used surrogate in epidemiological studies. However, the TyG index performed better in assessing insulin resistance and allowing earlier identification of the development of diabetes and its complications than HOMA-IR regardless of the use of insulin-related medication and diabetes status (18–21). Few studies have been conducted to evaluate the association between osteocalcin and insulin resistance assessed by the TyG index. Therefore, we aimed to investigate the effect of N-MID on the risk of exacerbating the level of insulin resistance in a T2D population.

## Materials and methods

### Subjects

This was a retrospective cross-sectional study. Men with T2D hospitalized in the Department of Endocrinology at the Fifth Affiliated Hospital of Sun Yat-sen University (Zhuhai, Guangdong, China) from January 2020 to May 2022 were recruited. The protocol was approved by the Ethics Institutional Review Board of the Fifth Affiliated Hospital of Sun Yat-sen University. Based on the 2010 criteria of the American Diabetes Association (ADA) (22), participants with fasting plasma glucose (FPG) ≥7.0 mmol/L or 2-h plasma glucose (2-h PG) ≥11.1 mmol/L during the 75-g oral glucose tolerance test (OGTT) or hemoglobin A1c (HbA1c)≥6.5% were diagnosed with T2D. Exclusion criteria for analysis included:

(1) Acute complications such as ketoacidosis and hyperosmolar coma;
(2) Severe infection;
(3) Hepatic dysfunction (serum level of aminotransferase was higher than three times the upper limit of normal);
(4) Renal failure including low eGFR (eGFR < 60 ml/min/1.73 m^2^) and renal transplant;
(5) Thyroid dysfunction or parathyroid disease;
(6) Recent history of fracture;
(7) Use of medications such as glucocorticoids, vitamin D, calcitonin and bisphosphonate;
(8) Participants with missing osteocalcin or TyG index measurements

### Anthropometric, clinical, and socio-demographic parameters

Patient data including age, the duration of diabetes, smoking status, alcohol consumption and medication use (lipid-lowering and hypoglycemic agents) were obtained from interviews at the initial study visit and clinical records. Smoking status was classified as ‘yes’ if the participants smoked daily or almost daily. Alcohol consumption was defined as ‘yes’ if the participants drank weekly or almost weekly. The anthropometric information of each participant was measured by the trained investigators. Body mass index (BMI) was calculated by dividing body weight (kg) by height in meters squared (m^2^). Blood pressure was measured with an automated electronic device in the seated position after resting for at least 5 min.

### Laboratory assays

Blood samples were collected following overnight fasting. Triglycerides (TG), total cholesterol (TC), high-density lipoprotein cholesterol (HDL-C), low-density lipoprotein cholesterol (LDL-C), alanine aminotransferase (ALT) and aspartate aminotransferase (AST) were determined by colorimetry. Fasting blood glucose (FBG) and glycated hemoglobin (HbA1c) were measured by using the hexokinase method and high-performance liquid chromatography respectively. N-terminal mid-fragment of osteocalcin (N-MID), the most stable form of osteocalcin in serum, and C-peptide were measured by an automated Roche electrochemiluminescence system. The TyG index was determined as ln(fasting TG [mg/dL] × FBG [mg/dL]/2). HOMA-IR was computed with FBG and C-peptide levels using the HOMA calculator v2.2.3 (https://www.dtu.ox.ac.uk/HOMACalculator/).

### Definitions

Insulin resistance is not an exclusively pathological concept, and it can also exist in physiological conditions such as adolescence, pregnancy, and aging (23–25). Therefore, due to the differences in race, gender, study population, and health status, criteria for assessing insulin resistance are often inconsistent, and establishing a universal normal range remains a problem. In previous studies, it is recommended that the upper quartile of insulin resistance (or the lower quartile of insulin sensitivity) of the specific population under a study be used as the cutoff for insulin resistance, regardless of the test method (26–28). As markers of insulin resistance, the higher the TyG index and HOMA-IR, the higher the level of insulin resistance. Thus, we defined severe insulin resistance as the TyG index and HOMA-IR above the 75th percentile values [IR: TyG index ≥75%tile (≥9.79); HOMA-IR≥75%tile (≥2.32)].

### Statistical analysis

The characteristics of the participants were described according to the overall and grouped by the tertiles of N-MID. The distribution of the data was assessed by the Shapiro-Wilk test and if the P value was < 0.05, data was considered to be non-normally distributed. Data were presented as mean ± standard deviation (SD) or median (interquartile range) for continuous variables and as the frequency (%) for categorical variables. Differences among N-MID groups were determined with the Kruskal Wallis test for continuous variables and the χ^2^ test for categorical variables.

We constructed binary logistic regression models to assess whether N-MID concentration (independent variable) was associated with the prevalence of increased TyG index or HOMA-IR (dependent variable), respectively treating N-MID as a continuous and categorical variable with participants in the lowest tertile of N-MID as the referent group. We performed tests for linear trend by entering the median values of N-MID tertile groups as a continuous variable in the models. Adjustments were performed in model 1: age, BMI and diabetes duration, and in model 2: age, BMI, diabetes duration, HbA1c, TC, hypertension, smoking status, the use of hypoglycemic agents and the use of lipid-lowering agents. Since the TyG index is itself composed of glucose and TG values, the glucose and triglyceride were not simultaneously included in the multivariable analysis as confounders. We further explored the potential non-linear association between N-MID (continuously measured) and the prevalence of severe insulin resistance assessed by the TyG index using a restricted cubic spline (RCS) model with three knots at the 10th, 50th, and 90th percentiles. In addition, we performed subgroup analysis and interaction testing on the association between N-MID and the level of insulin resistance quantified by the TyG index to evaluate possible modifications with full adjustment in model 2.

All analyses were performed using IBM SPSS Statistics 26 and R software (version 4.2.1). A 2-sided P < 0.05 was considered to indicate statistical significance.

## Results

### The demographic and clinical characteristics of the study population by N-MID tertiles

**Table 1** showed the demographic and clinical characteristics of the population according to the overall and grouped by N-MID tertiles. We recruited 667 subjects with T2D from the Department of Endocrinology at the Fifth Affiliated Hospital of Sun Yat-sen University. We used the Shapiro-Wilk test for data normality, for the reason that sample size of the present study was 667 and Royston et al. (29) have produced an extension to the Shapiro-Wilk test allowing sample sizes up to 2000, which has been recommended as the numerical means for assessing data normality. The results showed that all continuous variables did not fulfill the normality assumption. Therefore, continuous variables were presented as median (interquartile range) in **Table 1**. The median age of the study participants was 54.0 (45.0-62.0), and the median N-MID was 11.60 (9.13-14.50) ng/mL. The N-MID range for each tertile was 2.24-9.88 ng/mL, 9.89-13.5 ng/mL and 13.5-36.9 ng/mL. Patients in first tertile of N-MID were elder compared to the second tertile and the third tertile. There were no statistically significant differences in BMI, ALT and AST among N-MID tertiles (all P > 0.05). In the highest N-MID tertile, FBG and HbA1c were significantly lower than that in the middle N-MID tertile and the lowest N-MID tertile. For C-peptide, a significant difference was found only between the tertile 2 and tertile 3 of N-MID. In the lipid profile, there was a significant difference in the level of TC between the lowest and middle tertile of N-MID; significant differences in the level of LDL-C between the tertile 1 with tertile 2 and tertile 3 of N-MID; and no statistically significant differences in TG and HDL-C among the three groups. The TyG index in the highest tertile of N-MID was significantly lower than that in the middle tertile and the lowest tertile of N-MID. However, the HOMA-IR in the middle group was the lowest. Diabetes duration, hypertension, smoking status, the use of hypoglycemic agents and the use of lipid-lowering agents were significantly different among the three groups.

**Table 1:** Clinical characteristics of the study population according to the tertiles of N-MID.

Variables	Overall	Tertile 1	Tertile 2	Tertile 3	P value
N	667	223	222	222	
**N-MID (ng/mL)**	11.60 (9.13-14.50)	8.27 (7.24-9.15)	11.60 (10.72-12.69)	16.35 (14.48-18.80)	
**Age (years)**	54.0 (45.0-62.0)	57.0 (48.0-64.0)	53.5 (45.0-62.0)	51.0 (42.8-60.3)	**<.001**
Diabetes duration					<.001
**≤ 5 years**	395 (59.2)	99 (44.4)	141 (63.5)	155 (69.8)	
**> 5 years**	272 (40.8)	124 (55.6)	81 (36.5)	67 (30.2)	
**BMI (kg/m2)**	24.58 (22.77-26.64)	24.91 (22.82-27.12)	24.61 (23.01-26.79)	24.22 (22.49-26.31)	**.093**
**FBG (mmol/L)**	7.70 (6.31-10.33)	7.80 (6.46-10.92)	8.43 (6.64-11.14)	7.17 (5.90-8.96)	**<.001**
**HbA1c (%)**	8.7 (7.0-10.9)	8.7 (7.4-11.5)	9.5 (7.5-11.3)	7.8 (6.5-10.4)	**<.001**
**C-peptide (pmol/L)**	629.0 (423.5-862.5)	644.0 (432.5-854.0)	579.6 (405.0-811.5)	659.0 (443.8-937.0)	**.030**
**TG (mmol/L)**	1.55 (1.03-2.36)	1.58 (1.01-2.63)	1.57 (1.06-2.48)	1.47 (1.01-2.15)	**.322**
**TC (mmol/L)**	4.69 (3.95-5.49)	4.45 (3.69-5.42)	4.88 (4.15-5.63)	4.77 (3.95-5.43)	**.008**
**HDL-C (mmol/L)**	1.00 (0.86-1.16)	1.00 (0.84-1.17)	0.99 (0.85-1.15)	1.00 (0.89-1.17)	**.450**
**LDL-C (mmol/L)**	2.87 (2.12-3.53)	2.56 (1.83-3.35)	3.00 (2.36-3.58)	2.93 (2.20-3.63)	**<.001**
**ALT (U/L)**	20.00 (14.50-28.90)	19.70 (14.13-28.53)	20.30 (14.00-30.35)	20.00 (14.83-28.95)	**.704**
**AST (U/L)**	17.10 (14.20-22.00)	17.15 (14.50-22.00)	17.00 (14.00-22.25)	17.25 (14.20-22.10)	**.891**
**TyG index**	9.21 (8.65-9.79)	9.22 (8.67-9.88)	9.31 (8.79-9.88)	9.10 (8.51-9.61)	**.005**
**HOMA-IR**	1.75 (1.23-2.32)	1.85 (1.29-2.33)	1.62 (1.17-2.12)	1.84 (1.25-2.42)	**.022**
**Hypertension**	221 (33.1)	95 (42.6)	66 (29.7)	60 (27.0)	**.001**
**Smoking status**	257 (38.5)	69 (30.9)	91 (41.0)	97 (43.7)	**.014**
**Drinking status**	140 (21.0)	42 (18.8)	52 (23.4)	46 (20.7)	**.490**
**Hypoglycemic agents**	391 (58.6)	154 (69.1)	115 (51.8)	122 (55.0)	**<.001**
**Lipid-lowering agents**	128 (19.2)	62 (27.8)	35 (15.8)	31 (14.0)	**<.001**

All continuous variables are non-normally distributed and expressed as median (interquartile range). Categorical variables are expressed as n (%).

N-MID, N-terminal osteocalcin; BMI, body mass index; FBG, fasting blood glucose; HbA1c, glycated hemoglobin; TG, triglyceride; TC, total cholesterol; HDL-C, high-density lipoprotein-cholesterol; LDL-C, low-density lipoprotein-cholesterol; ALT, Alanine aminotransferase; AST, Aspartate aminotransferase; TyG, index triglyceride-glucose index; HOMA-IR, homeostasis model assessment for insulin resistance. The bold values denote significant difference among three groups using a chi-square test.

### Associations of N-MID with the prevalence of severe insulin resistance assessed by the TyG index and HOMA−IR

**Figure 1:**
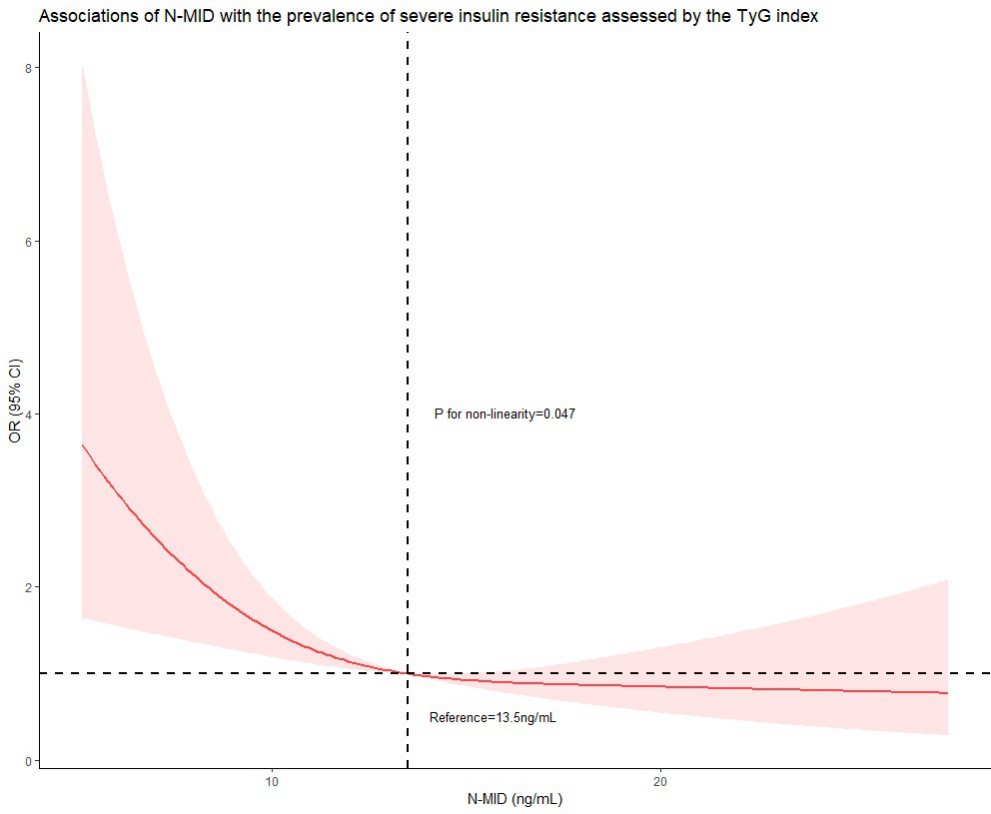
Restricted cubic spline curve was carried out with 3 knots at the 10th, 50th, and 90th percentiles of N-MID. When N-MID was approximately 13.5 ng/mL, as the reference, the estimated OR is equal to 1. The solid line represented point estimation on the association of N-MID (continuously measured) with the prevalence of severe insulin resistance assessed by the TyG index, and the shaded portion represented 95% CI estimation. Covariates in the model included age, BMI, diabetes duration, HbA1c, TC, hypertension, smoking status, the use of hypoglycemic agents and the use of lipid-lowering agents.

**Table 2** shows that there is a significant negative association between the concentration of N-MID and the prevalence of increased insulin resistance assessed by the TyG index in all models. When treating N-MID as a continuous variable, for each additional unit of N-MID, the proportional odds of having increased TyG index was 7.2% lower (OR=0.928, 95% CI 0.880–0.977, P = 0.005) after full adjustment. However, the concentration of N-MID is not related to HOMA-IR. We also showed ORs for the probability of increased insulin resistance assessed by the TyG index and HOMA−IR among tertile groups of N-MID with the lowest tertile group as the reference. The highest N-MID group showed a significantly lower risk of being in the high TyG index level compared to the lowest N-MID group (OR = 0.579, 95% CI 0.367-0.913) (Unadjusted model). After adjusting for age, BMI and diabetes duration, the risk of being in the higher TyG index level was still significantly lower (OR = 0.389, 95% CI 0.235-0.643) (Model 1). This association remained significant even after further adjusting for HbA1c, TC, hypertension, smoking status, the use of hypoglycemic agents and the use of lipid-lowering agents (OR = 0.507, 95% CI 0.290-0.885) (Model 2). However, compared to the lowest N-MID group, all the middle N-MID group was not related with the prevalence of severe insulin resistance measured by the TyG index. The prominent association of N-MID with the probability of increased insulin resistance assessed by HOMA-IR was not observed in all binary logistic regression models. RCS (**Figure 1**) showed that there was a nonlinear relationship between N-MID and the increased TyG index (P for non-linear association = 0.047). The prevalence of severe insulin resistance decreased with increasing N-MID until N-MID reached ~13.5 ng/mL. When N-MID is higher than 13.5 ng/mL, the risk of severe insulin resistance reached a plateau.

**Table 2:** ORs (95% CI) for the N-MID associated with severe insulin resistance assessed by the TyG index and HOMA-IR.

	Unadjusted		Model 1		Model 2	
	OR (95% CI)	P value	OR (95% CI)	P value	OR (95% CI)	P value
TyG index						
**N-MID**	0.947 (0.908, 0.987)	0.010	0.909 (0.865, 0.954)	0.000	0.928 (0.880, 0.977)	0.005
**Tertile 1**	Reference		Reference		Reference	
**Tertile 2**	1.125 (0.744, 1.701)	0.578	0.899 (0.575, 1.405)	0.640	0.789 (0.478, 1.303)	0.354
**Tertile 3**	0.579 (0.367, 0.913)	0.019	0.389 (0.235, 0.643)	<0.001	0.507 (0.290, 0.885)	0.017
**P for trend**	0.014		<0.001		0.015	
HOMA-IR						
**N-MID**	1.017 (0.978, 1.058)	0.386	1039 (0.994, 1.087)	0.088	1.030 (0.984, 1.079)	0.203
**Tertile 1**	Reference		Reference		Reference	
**Tertile 2**	0.682 (0.410, 1.132)	0.138	0.673 (0.384, 1.179)	0.167	0.673 (0.379, 1.195)	0.176
**Tertile 3**	1.293 (0.812, 2.060)	0.279	1.593 (0.937, 2.709)	0.085	1.545 (0.882, 2.704)	0.128
**P for trend**	0.165		0.036		0.061	

Odds ratio (OR) 95% confidence interval (CI).

Model 1: adjusted for variables age, BMI and diabetes duration.

Model 2: adjusted for variables in model 1 plus HbA1c, TC, hypertension, smoking status, the use of hypoglycemic agents and the use of lipid-lowering agents.

### Subgroup analysis for associations between N-MID and the prevalence of severe insulin resistance assessed by the TyG index

**Figure 2:**
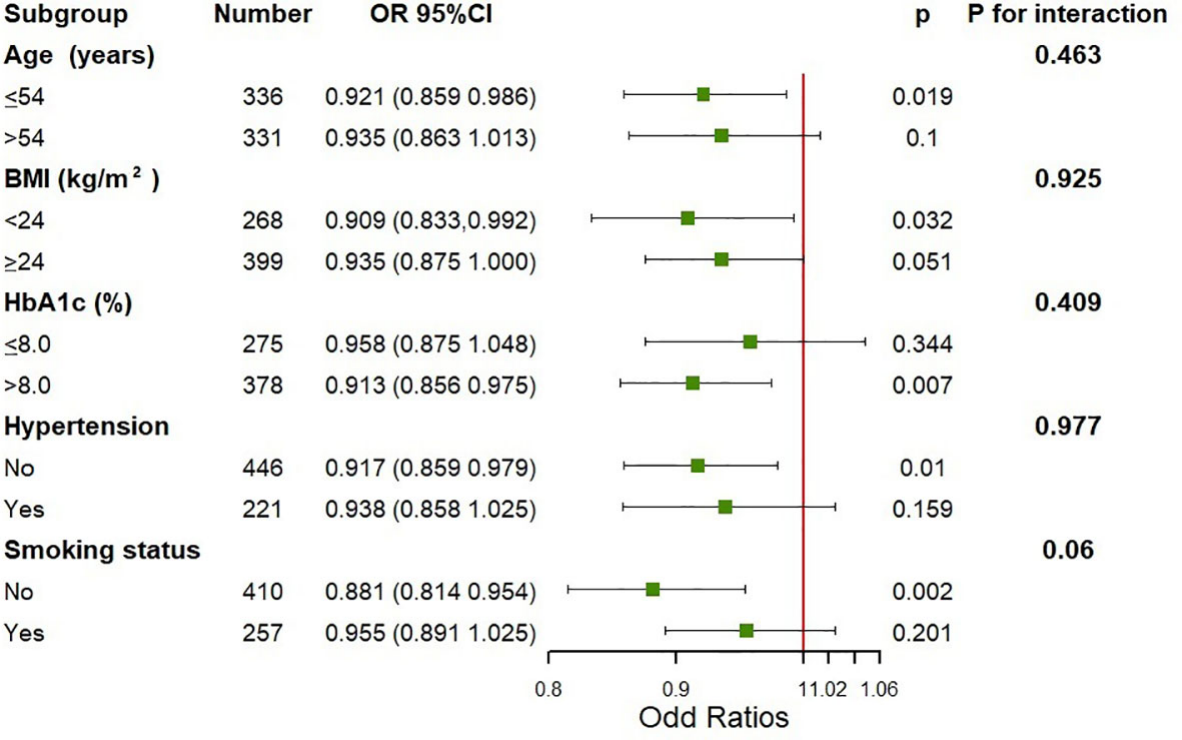
Subgroup analysis on the association between N-MID and the prevalence of severe insulin resistance assessed by the TyG index. Adjusted for age, BMI, diabetes duration, HbA1c, TC, hypertension, smoking status, the use of hypoglycemic agents and the use of lipid-lowering agents, if not be stratified.

Additionally, we conducted the subgroup analysis of the association between N-MID and the prevalence of increased insulin resistance assessed by the TyG index using binary logistic regression models stratified by age, BMI, HbA1c, the history of hypertension and smoking status (**Figure 2**). The models were fully adjusted for anthropometric, clinical, and socio-demographic variables, if not be stratified. There was no significant association between N-MID and increased TyG index level in men older than 54 years, whose HbA1c was lower than 8%, those having a history of hypertension, and those who smoked (all P ≥ 0.05). On the other hand, the N-MID was significantly associated with a lower prevalence of severe insulin resistance in a subgroup of those younger than 54 years (OR = 0.921, 95% CI 0.859, 0.986), those with BMI lower than 24 kg/m^2^ (OR= 0.909, 95% CI 0.833, 0.992), those HbA1c higher 8% (OR =0.913, 95% CI 0.856, 0.975), those without a history of hypertension (OR = 0.917, 95% CI 0.859, 0.979) and those without a history of smoking (OR = 0.881, 95% CI 0.814, 0.954) (all P < 0.05). No interactions were detected in the stratified analysis.

## Discussion

This research investigated the relationship between osteocalcin and the prevalence of severe insulin resistance in Chinese men with T2D. The findings showed that the prevalence of severe insulin resistance was significantly associated with N-MID after adjusting for age, BMI, HbA1c, TC, hypertension, smoking status, the use of hypoglycemic agents and the use of lipid-lowering agents. However, the relationship was only observed when the TyG index was used to quantify the level of insulin resistance, and not when HOMA-IR was used. Additionally, a non-linear relationship was found between N-MID and the risk of increased TyG index. To the best of our knowledge, this is the first study to show a relationship between N-MID and the prevalence of severe insulin resistance assessed by the TyG index.

Insulin resistance is a primary risk factor for T2D (30). It could be an important strategy to improve insulin resistance for the prevention and treatment of diabetes and its complications. Osteocalcin is a peptide synthesized by mature osteoblasts and used as a biochemical marker for bone formation. Recently some basic studies have proved that osteocalcin could be an endocrine regulator of glucose metabolism, by enhancing pancreas islets cell proliferation, increasing insulin secretion, and improving insulin resistance (7), which makes osteocalcin possible to perform as a drug for alleviating insulin resistance and metabolic abnormalities if clinical studies come to the same conclusion. However, in clinical studies, the correlation between osteocalcin and insulin secretion or insulin resistance was still controversial. In this study, we found that osteocalcin had no relationship with HOMA-IR which is consistent with previous studies (9–12), but others thought that osteocalcin was inversely associated with HOMA-IR (13–15). A further novel finding in the present study is that when using the TyG index to quantify insulin resistance, there is a negative correlation between osteocalcin and insulin resistance, making our research results seem contradictory. However, our study also finds that osteocalcin is related to biochemical indicators of glucose metabolism including fasting glucose, HbA1c, and fasting C-peptide, which is in accordance with the conclusions of animal and cell experiments. Therefore, we are more inclined to the conclusion that in humans, there is a negative correlation between osteocalcin with glucose metabolism and insulin resistance. We then also used the method of the RCS model to further confirm that the TyG index decreases with increasing N-MID.

In addition, our conclusions could be illustrated by that each of insulin resistance methods has distinct advantages and limitations. To date, a wide variety of methods and indicators used to assess the β-cell function and insulin resistance have been developed, based on static, dynamic tests and calculations of their results. The HEC method, measuring insulin resistance/sensitivity directly, is the gold standard method (31). But it is not available and practical in clinical practice because it is laborious and time-consuming. HOMA-IR using glucose and fasting insulin/C-peptide in the fasting state has been the most widely used surrogate in clinical practice and epidemiological studies due to its convenience (32). Unfortunately, HOMA-IR is the least accurate, partly because of the lack of standardization of insulin immunoassays (33), which may have led to inconsistent conclusions in previous studies analyzing the relationship between osteocalcin and insulin resistance. The TyG index, a simple index to evaluate insulin resistance can be widely used because the assay of glucose and triglycerides is common in clinical practice and available in all clinical laboratories. What’s more, quantification of insulin levels is not necessary, which means that the influence of insulin-related hypoglycemic agents is eliminated. Therefore, the TyG index is a better surrogate to assess insulin resistance compared with HOMA-IR, proved by the studies founding that the TyG index is stronger associated with the HEC (19) and better predicts the prevalence of T2D and its complications (21, 34).

In this study, we were unable to elucidate the exact mechanism of why the level of N-MID was associated with the TyG index rather than HOMA-IR in patients with T2D. Firstly, considering that serum insulin levels are susceptible to interference by proinsulin, insulin antibody and exogenous insulin, patients with diabetes, whether they are insulin-using or not, were usually tested for fasting C-peptide only, resulting in no available fasting insulin data in the present study. The HOMO-IR calculated from C-peptide may not be comparable with those calculated from insulin value (35). Secondly, it may be ascribed to the metabolism of glucose and insulin being made through a sophisticated process related to diverse stimuli in several tissues such as the liver, adipocytes, and skeletal muscles. Note that the severity of insulin resistance in these tissues may vary depending on inherited risk factors, lifestyle factors, and ethnicity (36, 37). In particular, insulin resistance in the liver and skeletal muscle deserves attention for a better understanding and appropriate intervention in T2D, which have been depicted as central insulin resistance and peripheral insulin resistance, respectively. Because the concentration of fasting plasma glucose mainly depends on the rate of hepatic glucose production, and insulin is the primary regulator of hepatic glucose production, HOMA-IR principally reflects the insulin resistance of the liver (38). On the other hand, it is recognized that the TyG index specifically reflects muscle-related insulin resistance, the explanation of which may be that increased triglyceride level in the blood tends to suppress insulin activity in muscle and interfere with glucose uptake (39). It is worth noting the estimate of the contribution of skeletal muscle to the insulin-stimulated removal of glucose from the blood was approximately 70% during HEC in humans (40), indicating that skeletal muscle is a primary organ responsible for systemic glucose homeostasis. Therefore, the different associations between N-MID with the TyG index rather than HOMA-IR in patients with T2D may be due to the direct and indirect mechanisms by which osteocalcin affects glucose metabolism. Osteocalcin could increase β cell proliferation, improve insulin expression, and regulate peripheral insulin resistance *via* adiponectin, an adipocyte-specific insulin-sensitizing hormone (7). What’s more, osteocalcin can also directly increase the glycogen and fatty acid catabolism in muscle fibers by inducing the translocation of the glucose transporter, GLUT4, to the plasma membrane (41), and increasing the expression of interleukin-6 expression in muscle, a myokine who promotes glucose uptake and fatty acid oxidation in muscle (42).

To the best of our knowledge, there has been no report thus far about the association between osteocalcin with insulin resistance assessed by the TyG index. However, there are several limitations in this study: (1) Insulin resistance was assessed by surrogate markers, HOMA-IR and TyG index, instead of the gold standard, the HEC test. However, accumulating evidence has validated the sensitivity and specificity of the TyG index (18). (2) We analyzed only male subjects with T2D hospitalized at the Fifth Affiliated Hospital of Sun Yat-sen University. Therefore, the participants who were enrolled in this research might have relatively severe states of health. More importantly, the correlation between osteocalcin levels and glucose metabolism appears to be gender-dependent. The lack of female subjects, our results might be different from those in which female populations were included. (3) We cannot clarify the cause-effect between osteocalcin and glucose and lipid metabolism because of its cross-sectional nature. (4) We didn’t include bone mineral density (BMD) in the multivariate adjustment, which was known to be associated with insulin resistance and diabetes (43). However, to minimize the confounding effect of bone health, we have excluded patients with a recent fracture or who are on medications, such as vitamin D and bisphosphonate, for pre-existing osteoporosis.

In conclusion, we demonstrate that serum osteocalcin levels are independently associated with the new surrogate of insulin resistance, the TyG index in men with T2D, reflecting that osteocalcin mainly regulates peripheral insulin resistance. Additional investigations and further confirmation are needed to prove the potential of osteocalcin as a drug for alleviating insulin resistance and metabolic abnormalities.

## Data availability statement

The raw data supporting the conclusions of this article will be made available by the authors, without undue reservation.

## Ethics statement

The studies involving human participants were reviewed and approved by the Ethics Institutional Review Board of the Fifth Affiliated Hospital of Sun Yat-sen University. The ethics committee waived the requirement of written informed consent for participation.

## Author contributions

HH and YZ reviewed the literature and conceived the study. YZ was in charge of overall direction and planning. HH and AW were involved in patient selection, sample, data collection, measurements and statistical analysis. HH interpreted the results and drafted the manuscript. YZ and LC reviewed and edited the manuscript. All authors contributed to the article and approved the submitted version.
